# Analysis of the Impact on the Mechanical Properties of Modification of Oligohydroxyethers in Organic Solvent Solution with Rubbers

**DOI:** 10.3390/polym13040519

**Published:** 2021-02-09

**Authors:** Vitalii Bezgin, Agata Dudek, Adam Gnatowski

**Affiliations:** 1 Department of Production Engineering and Materials Technology, Czestochowa University of Technology, 42-201 Czestochowa, Poland; vsbezgin@gmail.com; 2 Department of Technology and Automation, Czestochowa University of Technology, 42-201 Czestochowa, Poland; gnatowski@ipp.pcz.pl

**Keywords:** hydroxyeters, modification, rubbers, mechanical properties

## Abstract

This paper proposes and presents the chemical modification of linear hydroxyethers (LHE) with different molecular weights (380, 640, and 1830 g/mol) with the addition of three types of rubbers (polysulfide rubber (PSR), polychloroprene rubber (PCR), and styrene-butadiene rubber (SBR)). The main purpose of choosing this type of modification and the materials used was the possibility to use it in industrial settings. The modification process was conducted for a very wide range of modifier additions (rubber) per 100 g LHE. The materials obtained in the study were subjected to strength tests in order to determine the effect of the modification on functional properties. Mechanical properties of the modified materials were improved after the application of the modifier (rubber) to polyhydroxyether (up to certain modifier content). The most favorable changes in the tested materials were registered in the modification of LHE-1830 with PSR. In the case of LHE-380 and LHE-640 modified in cyclohexanol (CH) and chloroform (CF) solutions, an increase in the values of the tested properties was also obtained, but to a lesser extent than for LHE-1830. The largest changes were registered for LHE-1830 with PSR in CH solution: from 12.1 to 15.3 MPa for compressive strength tests, from 0.8 to 1.5 MPa for tensile testing, from 0.8 to 14.7 MPa for shear strength, and from 1% to 6.5% for the maximum elongation. The analysis of the available literature showed that the modification proposed by the authors has not yet been presented in any previous scientific paper.

## 1. Introduction

The analysis of the scientific and technical literature and the observed dynamically growing demand for polymeric materials indicates that there is currently a need to search for new polymeric materials or methods of their modification. The growing interest in new polymeric materials stimulates the search for new or improved properties of polymers that have been widely used in industry. This phenomenon has led to the rapid development of research on a variety of problems related to, e.g., methodologies of modification of polymeric materials. Furthermore, the modification of polymers is in many cases more economical than synthesizing new monomers and polymers. Nowadays, many important polymeric materials with wide application in industry are obtained through modification. There are two types of polymer modification: chemical and physical. Physical modification of polymers consists in a deliberate change of their functional properties through the action of physical factors, such as heat energy, ultrasound, electric field, magnetic field, or by introducing modifiers into the polymers, which leads to a change in the polymer composition. Chemical modification consists in changing the properties of polymers using chemical factors and can be conducted directly during the polymer synthesis or on the finished polymer. In the case of chemical modification conducted on the finished polymer, several chemical reactions are possible: substitution, exchange of functional groups, blocking of polar groups, and crosslinking [1,2,3,4,5,6,7,8,9]. Research on materials obtained by means of modification in solution and research of possibilities of production of modified composites based on these materials have been carried out in many studies [10,11,12].

The modification of industrial polymeric materials is currently the most promising field of modern chemistry of macromolecular compounds [6,7,8,9,10,11,12,13].

Linear hydroxyether (LHE) based on bisphenol A and epichlorohydrin was first synthesized at the end of 1930 by Kastan and Greenley. Nowadays, the production capacity of polyhydroxyethers is about 1,200,000 tons/year, which accounts for as much as 90–95% of the global production capacity [14].

Polyhydroxyethers have been widely used in engineering materials in the form of multicomponent setting materials containing, in addition to resin and hardener, solvents and plasticizers, active diluents and elasticity enhancers, fine and granular fillers, dyes and pigments, and other functional additives that give special properties, for example, electrical conductivity [1,2,3,4,5].

In practice, polyhydroxyethers are modified to improve their technological properties or reduce product costs. The main objective of the modification of polyhydroxyethers is to improve their strength, resistance to external destructive factors or resistance to active chemical environments [3,4,5].

The analysis of publications and patents related to hydroxyethers shows that the challenge that scientists are still facing is to find a new method to modify these materials, not only chemically cured but also thermoplastic. Especially the latter, due to their undeniable advantages (no need for using hardeners and the possibility of multiple processing), seem to be promising materials [3,4,5,6,7,8,9,13,14,15,16,17,18,19,20,21,22,23,24,25,26,27,28]. Analyzing the kinetic models of processes with the use and vulcanization of rubbers, a number of the most stable and tested polymer rubbers can be selected, such as polysulphide (PWK) and styrene-butadiene (SBR) rubbers. Many authors conduct research on the modification and properties of these materials [29,30,31,32,33].

This article discusses a new proposal for the chemical modification of chemically cured and thermoplastic hydroxyethers, which can be successfully used industrially. The modification is presented for a very wide range of both the materials used and the addition of modifiers. For modification, the following types of rubbers were applied: polychloroprene rubber (PCR), SBR, and polysulfide rubber (PSR), due to different structures and properties. The analysis of the available literature showed that the modification proposed by the authors has not yet been presented in any previous scientific paper.

## 2. Materials

The analysis of materials that can be used to modify polyhydroxyethers carried out during the study showed that rubbers are potential plasticizers of LHE and that their use allows for obtaining materials with improved functional properties. Three types of rubbers were selected for modification: PCR, SBR, and PSR, and the use of LHE with different molecular weights was planned. Dissolution of components was used as a modification method.

**Scheme 1:**
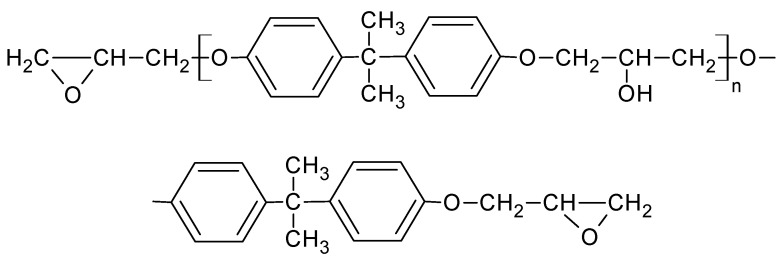
Chemical formula for polyhydroxyethers (LHE).

The chemical formula for polyhydroxyethers (LHE) is reported in Scheme 1.

**Scheme 2:**
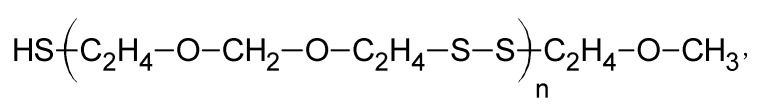
Chemical formula for polysulfide rubber.

**Scheme 3:**
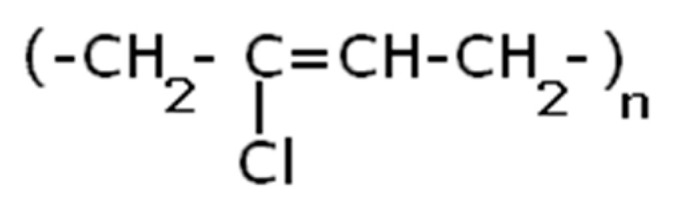
Chemical formula for polysulfide rubber.

**Scheme 4:**
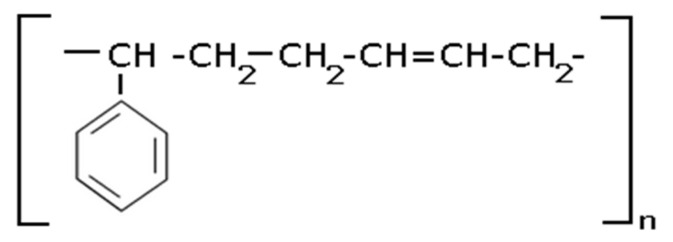
Chemical formula for styrene-butadiene rubber.

The modifier was:

- Polysulfide rubber (PSR) in a liquid form, with a viscosity of 28 Pa*s with the content of sulfhydryl groups of 3.1%, where (n = 6–23). The formula of polysulfide rubber is shown in Scheme 2.

- PCR—polychloroprene rubber (shown in Scheme 3).

- SBR—styrene-butadiene rubber (shown in Scheme 4).

Chemically cured and thermoplastic polyhydroxyethers (LHE) with molecular weights of 380, 640, and 1830 g/mol were selected for the study. They were chemically modified with the addition of various rubbers in the solution of organic solvents. The modification was performed using the following types of solvents:

- Trichloroethene (TCE);
- Dichloroethane (DHE);
- Chloroform (CF);
- Ethyl acetate (EA);
- Toluene (TL);
- Cyclohexanol (CH).

To the mentioned solvents, 5–20% vol. LHE was added (80–95% of the solvent and 5–20% of modified LHE per 100 mL of solution). The mixture was heated to 40 °C and mixed for 30 min. The possibility of dissolving hydroxyethers in the solvents used is presented in the form of Table 1.

**Scheme 5:**
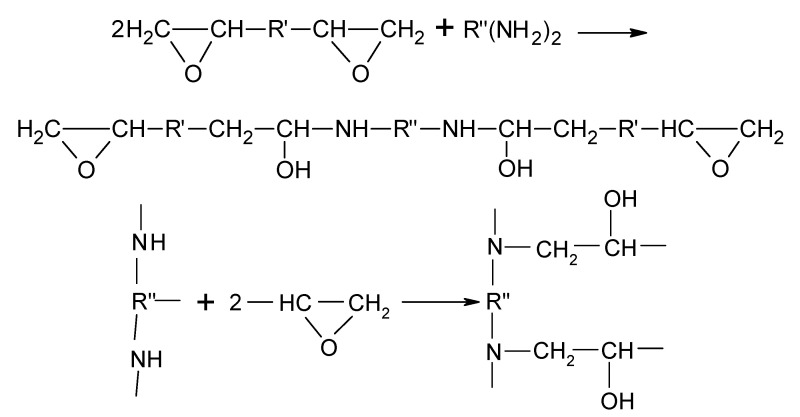
Curing process.

In order to obtain the solid phase, PEPA hardener (polyethylene polyamine) was added to LHE-380. The curing process took place according to the following pattern (Scheme 5).

**Scheme 6:**
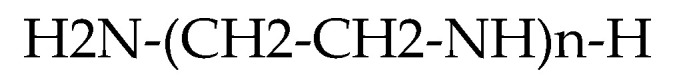
Chemical formula for of PEPA.

Based on the research [21,25], the following LHE curing procedure was applied: 5 g of PEPA hardener was added to 100 g of polymer at 25 °C, and then, after 15 min of mixing, the samples were formed using silicone molds. PEPA was used for curing LHE-380 (Scheme 6), where (n = 1–5).

From Table 1, it can be seen that only in the case of two solvents (CH and CF), it was possible to dissolve 20% wt. LHE in the solvent. Therefore, two solvents were selected to modify LHE: CH and CF.

The modification process was conducted for a very wide range of modifier additions (rubber) per 100 g LHE.

## 3. Research Methodology

The materials obtained in the study were subjected to strength tests in order to determine the effect of the modification on functional properties. The results of mechanical properties are the arithmetic means of five measurements, and the standard deviation did not exceed 5%.

Compressive strength tests were performed in accordance with ISO 604—Plastics—Determination of compressive properties. The sample for the compressive strength testing was in the form of a 5 mm × 5 mm × 5 mm cube. The samples were formed using a silicone mold.

Tensile properties were tested in accordance with the requirements of ISO 527-1—Plastics—Determination of tensile properties, Part 1: General principles, and ISO 527-2—Plastics—Determination of tensile properties, Part 1: General principles. Flat paddle-shaped samples were used for the tests. Samples were formed by placing the material in a silicone mold. The sample thickness was 4.0 ± 0.2 mm, the width of the measurement part was 10 ± 0.2 mm, and the total sample length was 60 mm. Furthermore, during the static tensile test, the relative elongation at the highest tensile stress was determined for the materials tested.

A shear strength test of a polymer/metal connection (steel S235) was conducted as follows: after application of the polymeric material on previously cleaned and degreased (using acetone) working surfaces, the strength test of overlapping joints was performed for the elements glued together stretched in the adhesive plane according to ASTM D1002—Standard Test Method for Apparent Shear Strength of Single-Lap-Joint Adhesively Bonded Metal Specimens by Tension Loading (Metal-to-Metal) (ASTM D3165—Standard Test Method for Strength Properties of Adhesives in Shear by Tension Loading of Single-Lap-Joint Laminated Assemblies).

## 4. Results and Discussion

### 4.1. Analysis of Mechanical Properties of LHE-380 Modified with PSR, PCR, and SBR in CH Solution

**Figure 1:**
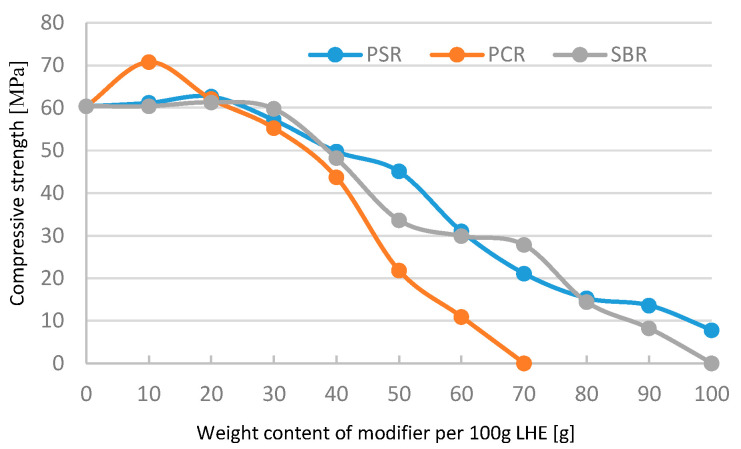
Compressive strength of linear hydroxyethers (LHE) 380 modified in CH solution.

The modification of LHE-380 in CH solution resulted mostly in a decrease in compressive strength with the increasing content of modifiers added to LHE-380 (Figure 1). Adding 10 g of PCR to 100 g of LHE-380 resulted in an increase of 10 MPa.

**Figure 2:**
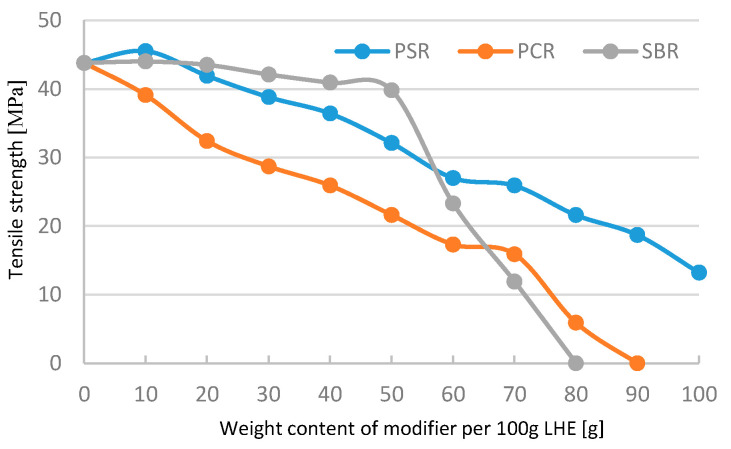
Tensile strength of LHE-380 modified in CH solution.

The modification of LHE-380 in CH solution adversely affected the tensile properties of the material (Figure 2). The observation of the materials obtained indicates that adding over 70 g of PCR and SBR modifiers to 100 g of LHE yields brittle materials.

**Figure 3:**
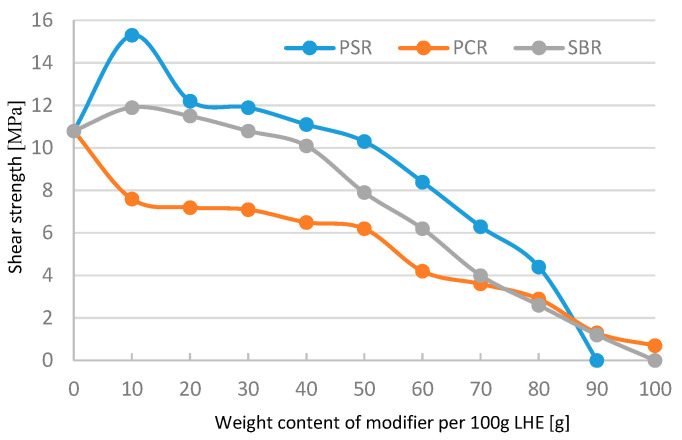
Shear strength of LHE 380 modified in CH solution.

**Figure 4:**
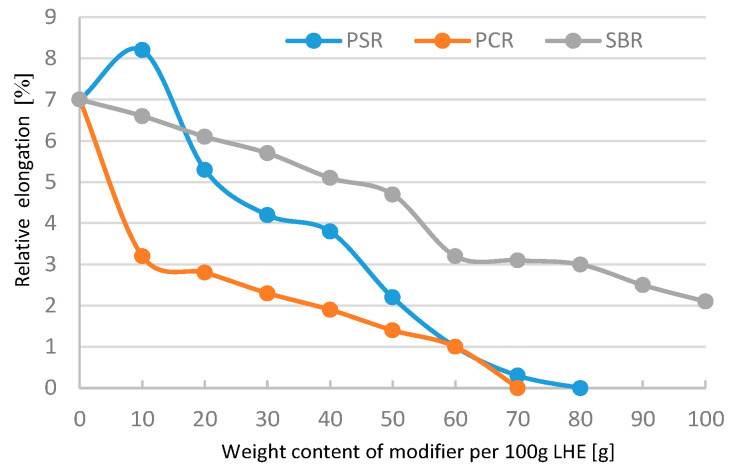
Relative elongation of LHE-380 modified in CH solution.

The addition of 10–30 g of PSR and SBR modifiers had a small effect on the increase in shear strength of materials (Figure 3). A further increase in the contents of PSR, SBR, and PCR modifiers led to a decrease in shear strength compared to LHE-380 without modification. The modification of LHE-380 in the CH solvent reduced the plasticity of the materials tested (Figure 4).

### 4.2. Testing of Mechanical Properties of LHE-380 Modified with PSR, PCR, and SBR in CF Solution

**Figure 5:**
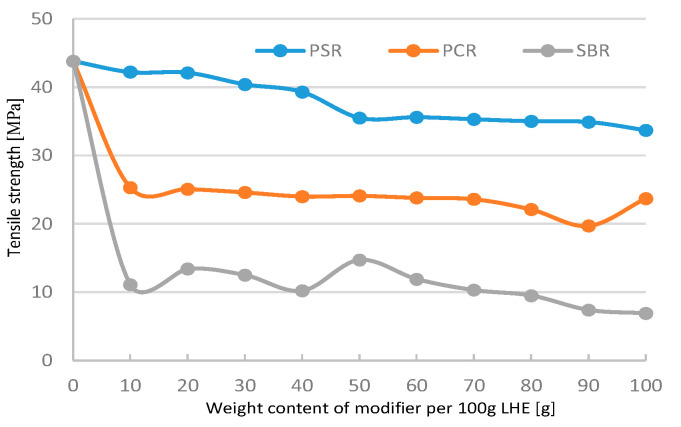
Compressive strength of the LHE-380 modified in CF solution.

**Figure 6:**
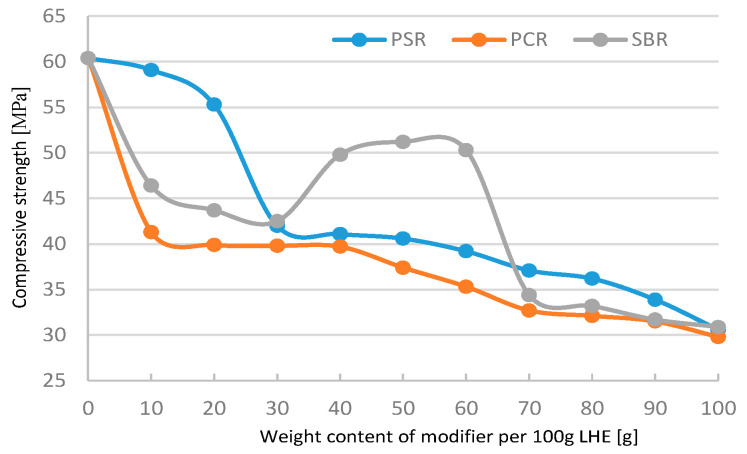
Tensile strength of LHE-380 modified in CF solution.

**Figure 7:**
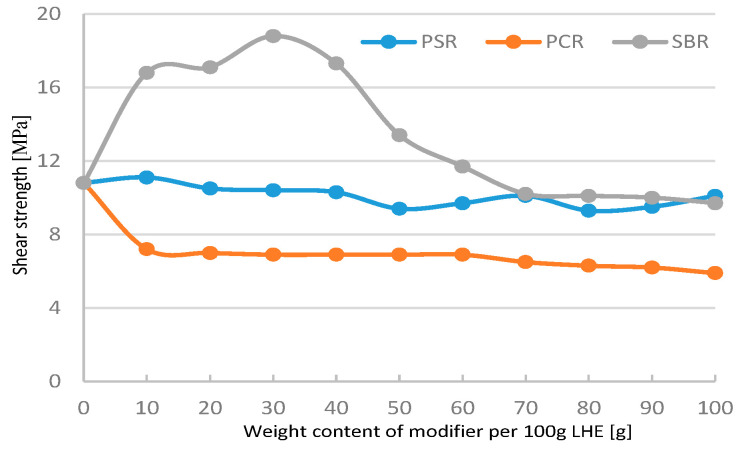
Shear strength of the LHE-380 modified in CF solution.

**Figure 8:**
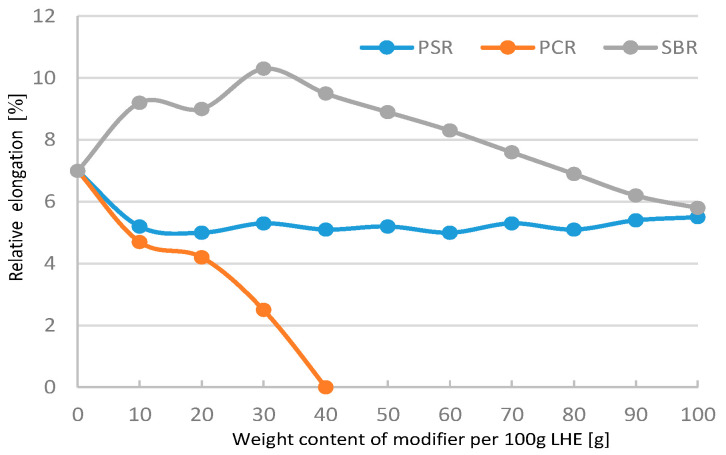
Relative elongation of LHE-380 modified in CF solution.

LHE-380 was modified in CF solvent solution, and then mechanical properties of the materials obtained were analyzed (see Figure 5, Figure 6, Figure 7 and Figure 8).

The modification of LHE-380 in CF solution (in the entire range of modifier content) affected the decrease in the compressive strength of the materials (Figure 5). An increase in compressive strength was observed for the content of 40–60 g of SBR. However, the results obtained were below those for the non-modified resin.

The analysis of tensile strength of LHE-380 modified in CF solution confirmed previous observations (Figure 6).

The best results for shear strength (Figure 7) were observed for adding SBR modifier to LHE-380. The modification of LHE-380 with SBR (Figure 8) also improved plasticity by up to 50%.

The use of modification by dissolving the components in the CF solvent did not improve the mechanical strength of the modified materials compared to LHE-380 without modification.

### 4.3. Testing of Mechanical Properties of LHE-640 Modified with PSR, PCR, and SBR in CH Solution

**Figure 9:**
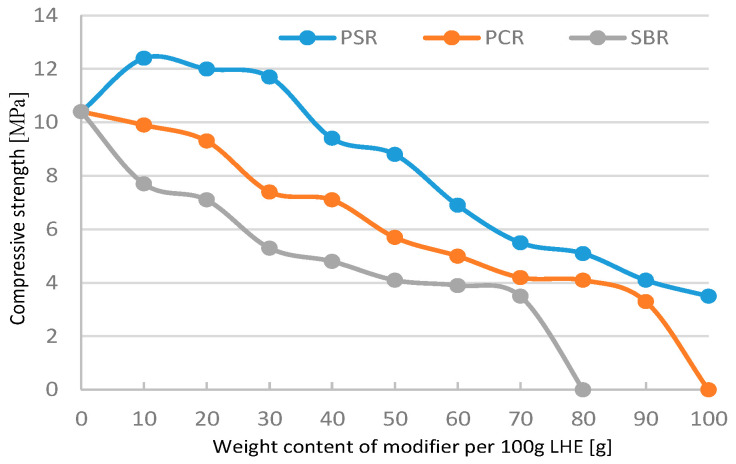
Compressive strength of the LHE-640 modified in CH solution.

**Figure 10:**
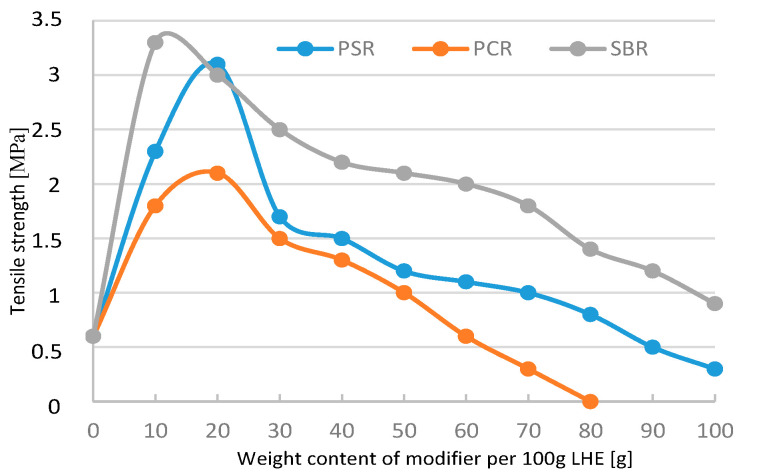
Tensile strength of LHE-640 modified in CH solution.

**Figure 11:**
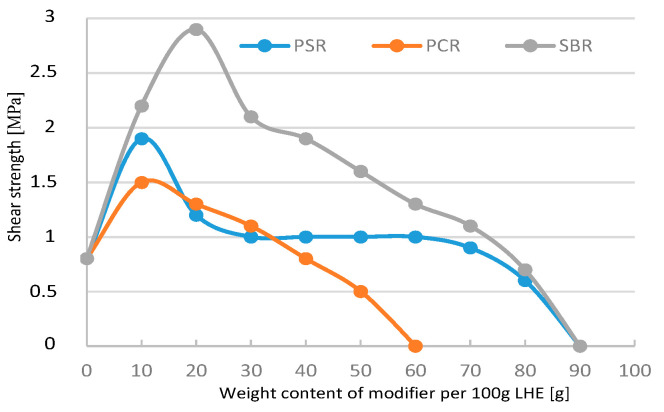
Shear strength of the LHE-640 modified in CH solution.

**Figure 12:**
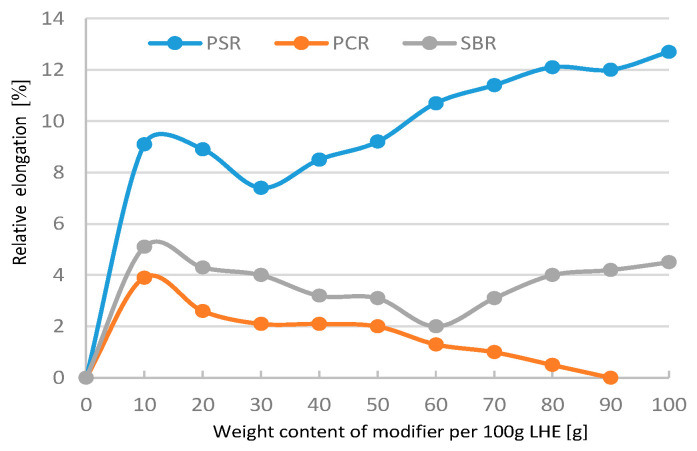
Relative elongation of LHE-640 modified in CH solution.

Thermoplastic resin LHE-640 was modified using rubbers in CH solution (Figure 9, Figure 10, Figure 11 and Figure 12).

The modification of CH solvent had a negative effect on the compressive strength of the modified material compared to LHE-640 resin without modification. An increase in compressive strength of materials can be obtained only if 10–30 g of PSR modifier is used (Figure 9).

The tensile strength of LHE-640 modified in CH solution increased up to 3.3 MPa when SBR and PSR were added and up to 2.1 MPa when PCR was used for modification (Figure 10). In the case of shear strength (Figure 11), the maximum increases in this parameter were 2.8 MPa for SBR, 1.9 MPa for PSR, and 1.5 MPa for PCR. The modification of LHE-640 in CH solution with a higher content of rubbers (over 60 g) had a negative effect on shear strength.

With a small addition of modifiers to LHE-640 in CH solution, the material showed an increase in relative elongation only when 10–20 g of modifier was used (Figure 12).

The modification of LHE-640 with rubbers in CH solution above a certain modifier limit led to a reduction in the strength properties of the materials, thus eliminating their wide application in industry.

### 4.4. Testing of Mechanical Properties of LHE-640 Modified with PSR, PCR, and SBR in CF Solution

**Figure 13:**
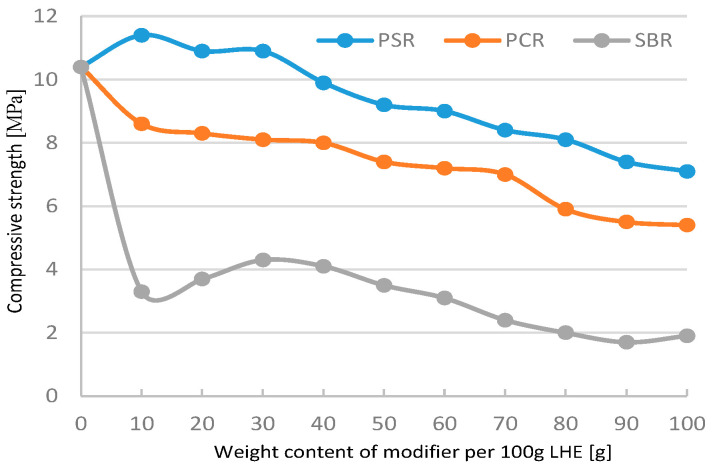
Compressive strength of LHE -640 modified in CF solution.

**Figure 14:**
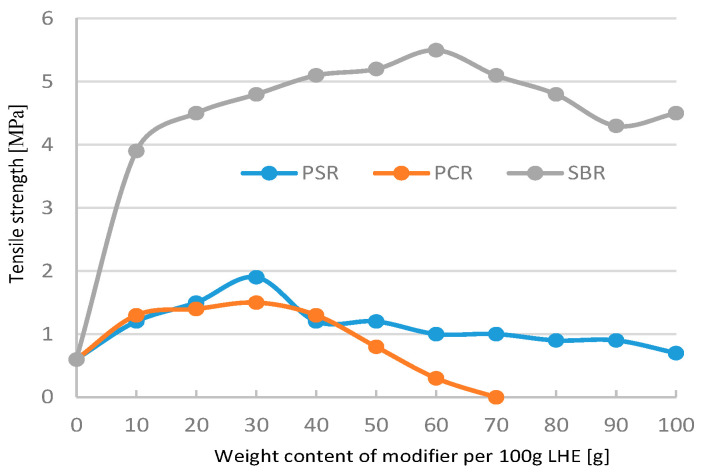
Tensile strength of LHE-640 modified in CF solution.

**Figure 15:**
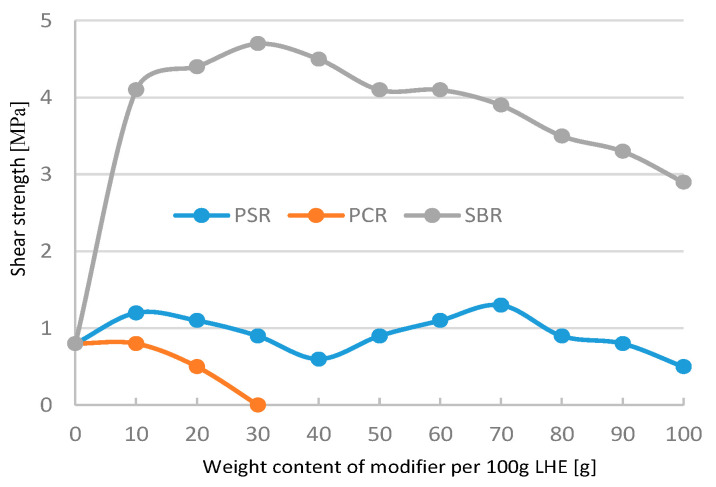
Shear strength of LHE -640 modified in CF solution.

**Figure 16:**
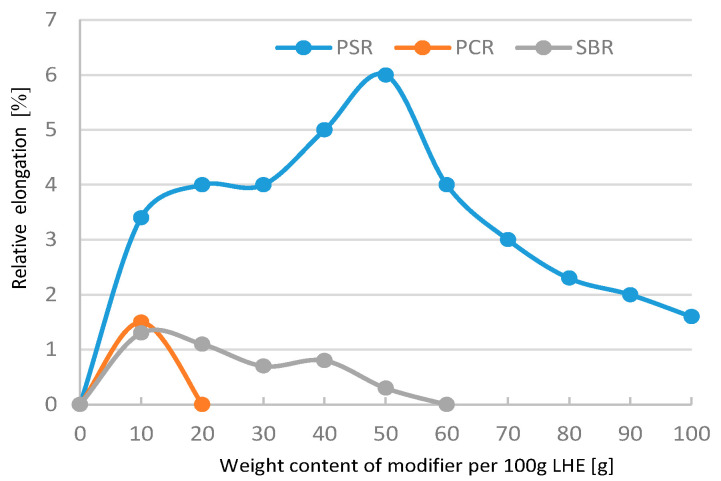
Relative elongation of LHE-640 modified in CF solution.

The modification of LHE-640 with rubbers in CF solution (Figure 13) had a negative effect on the compressive strength of the modified materials. The modification of LHE-640 (Figure 14) led to an increase in tensile strength from 0.6 to 5.5 MPa for SBR, 2 MPa for PSR, and 1.4 MPa for PCR. Shear strength increased from 0.8 to 4.5 MPa in the case of the addition of SBR and 1.3 MPa for PSR (Figure 15). The best results for LHE modification in terms of tensile elongation were obtained for PSR (Figure 16).

### 4.5. Testing of Mechanical Properties of LHE-1830 Modified with PSR in CH Solution

**Figure 17:**
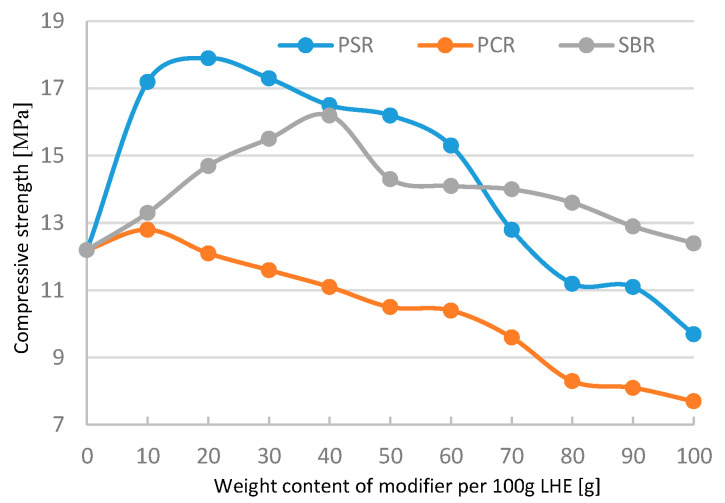
Compressive strength of LHE-1830 modified in CH solution.

**Figure 18:**
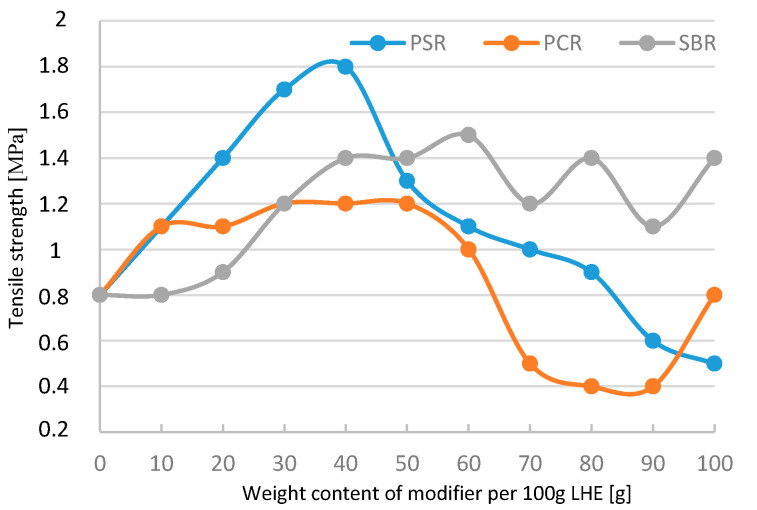
Tensile strength of LHE-1830 modified in CH solution.

**Figure 19:**
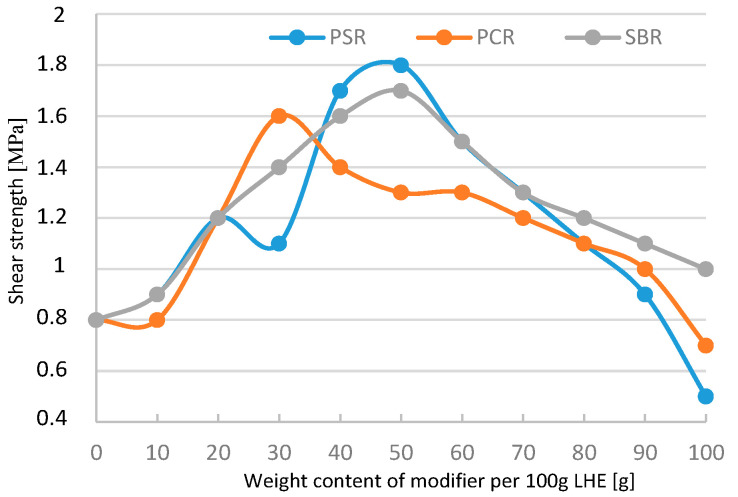
Shear strength of LHE-1830 modified in CH solution.

**Figure 20:**
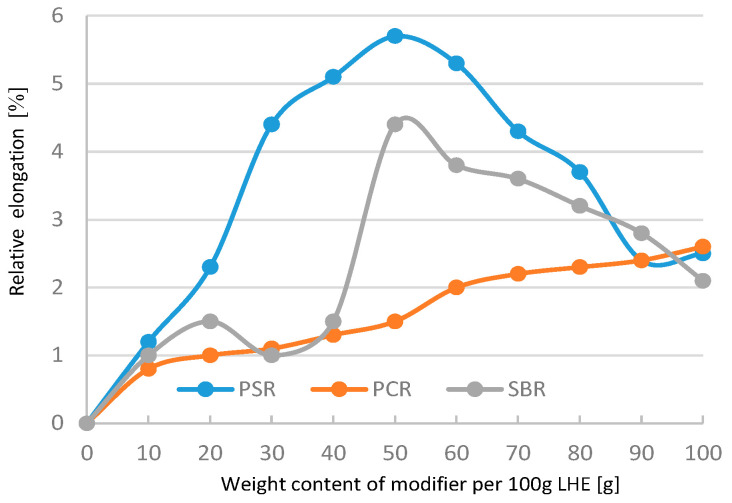
Relative elongation of LHE-1830 modified in CH solution.

In the case of the modification of LHE with a molecular weight of 1830 g/mol in CH solution, most results showed improvements in mechanical properties. The parameters increased from 12.1 to 15.3 MPa for compressive strength tests (Figure 17) and from 0.8 to 1.5 MPa for tensile testing (Figure 18). Shear strength increased from 0.8 to 14.7 MPa (Figure 19), whereas the maximum elongation increased from 1% to 6.5% (Figure 20). The analysis of the results of the modification of LHE with a molecular weight of 1830 g/mol in CH solution reveal that the use of this oligohydroxyether results in an increase in the functional properties of the polymer.

### 4.6. Testing of Mechanical Properties of LHE-1830 Modified with PSR in CF Solution

**Figure 21:**
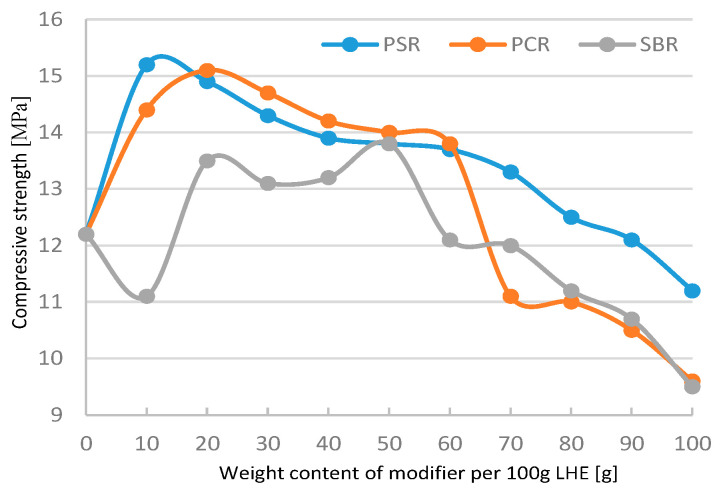
Compressive strength of the LHE-1830 modified in CF solution.

**Figure 22:**
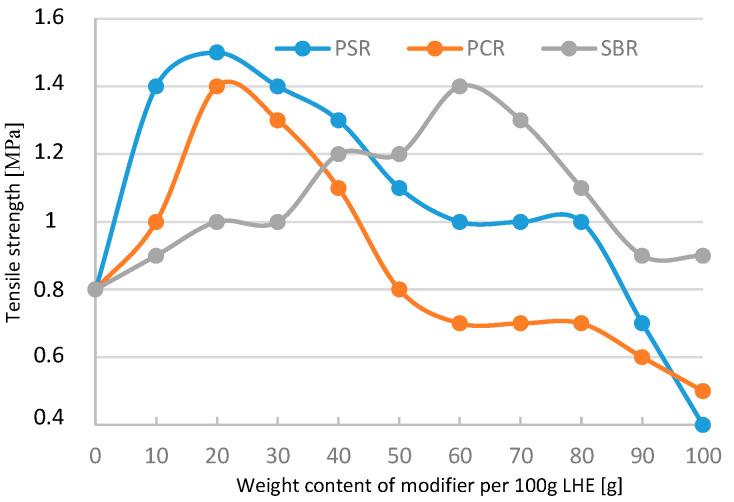
Tensile strength of LHE-1830 modified in CF solution.

**Figure 23:**
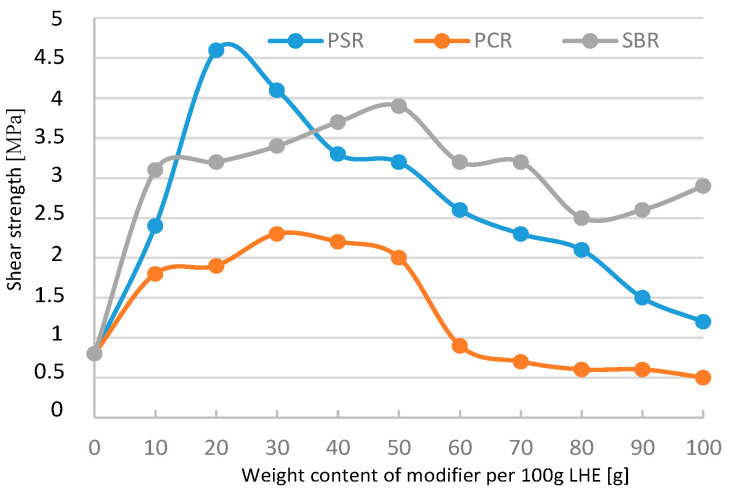
Shear strength of the LHE-1830 modified in CF solution.

**Figure 24:**
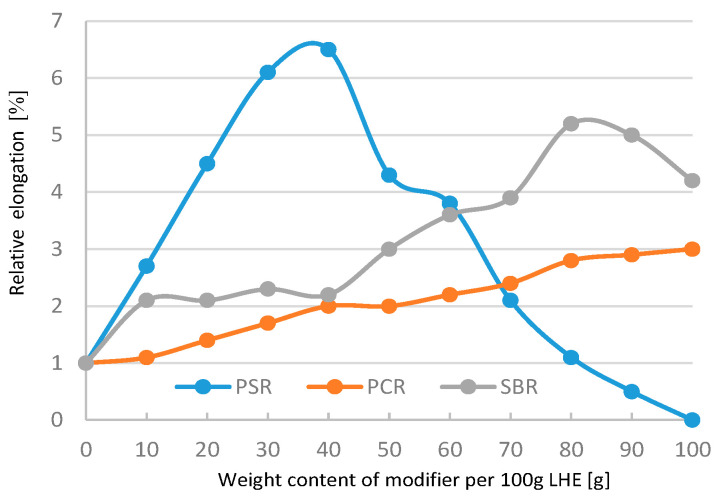
Relative elongation of LHE-1830 modified in CF solution.

The modification of LHE using PSR in CF solvent had a maximally positive effect on the compressive strength of the modified material compared to LHE-1830 resin without modification. An increase in compressive strength of materials can be obtained if 10–70 g of PSR modifier is added to 100 g (Figure 21). Tensile strength increased to a maximum of 1.8 MPa when PSR was added (Figure 22). In the case of shear strength (Figure 23), the maximum increase in this parameter was also up to 1.8 MPa. The addition of 10–60 g modifiers to 100 g of LHE-1830 in CF solution led to an increase in maximum elongation of up to 5.7% (Figure 24).

Significant changes of the tested materials were registered in the modification of LHE-1830 with PSR. The relationship of the modifier with the LHE caused the process of additional cross-linking of the LHE, which resulted in an increase in mechanical properties. In the case of LHE-1830 material, this increase can be better observed in the example of compressive and tensile strength. Lowering the value of the tested properties with increasing the amount of modifier added probably caused the phenomenon of breaking the linear structure of the LHE polymer.

## 5. Conclusions

The search for new materials for industrial applications has led to the rapid development of research on a variety of problems related to their modification. The latest research has been focused on the improvement of safety and economy of use, which is directly connected with the growing use of these materials in industry.

These arguments inspired the authors of the present paper to explore the problems of the modification of linear hydroxyethers (LHE) with different molecular weights using a method that is fully economically justified from the point of view of industrial application.

Three types of rubbers were selected for the modification of the polymeric matrix: polysulfide rubber (PSR), polychloroprene rubber (PCR), and styrene-butadiene rubber (SBR).

The modification of LHE with molecular weights of 380, 640, and 1830 g/mol in an organic solvent solution showed that the best properties to dissolve the components (among TCE, DHE, CF, EA, TL, ECL, and CH) were observed for CH and CF.

The modification of LHE-380 and LHE-640 in chloroform (CF) and cyclohexanol (CH) solvents did not lead to a significant improvement in the mechanical strength of the materials obtained.

Significant improvements in mechanical properties were observed in the case of the modification of LHE with a molecular weight of 1830 g/mol in CH and CF solutions.

Mechanical properties of the materials were improved by the addition of the modifier (mostly PSR rubber) to polyhydroxyethers (up to certain modifier content). This is probably due to the modifier interaction with the linear structure of LHE (interaction of functional groups in rubber with epoxy groups in LHE) and the occurrence of crosslinking between LHE polymer chains.

Too high a content of plasticizers in LHE adversely affected both the process efficiency and the properties of the materials obtained. The observed decrease in strength properties of the modified LHE (above the modifier limit value) is probably related to a decrease in the possibility of further crosslinking of the polymer.

## Figures and Tables

**Table 1:** Possibility to dissolve hydroxyethers in different solvents.

Hydroxyether	Solvents					
MW (g/mol)	TCE	DHE	CF	EA	TL	CH
380	20%	20%	20%	5%	5%	20%
640	5%	5%	20%	5%	5%	20%
1830	5%	5%	20%	5%	5%	20%

## Data Availability

Not applicable.
